# The state of hand rub dispensers in healthcare settings – a multicenter assessment in 19 German healthcare facilities

**DOI:** 10.1186/s13756-024-01470-w

**Published:** 2024-10-08

**Authors:** Christiane Herzer, Torsten Berg, Christine Hegemann, Tobias Gebhardt, Heide Niesalla, Christoph Senges

**Affiliations:** 1 GWA Hygiene GmbH, Stralsund, Germany; 2 BODE Chemie GmbH, HARTMANN group, Hamburg, Germany; 3 HARTMANN SCIENCE CENTER, BODE Chemie GmbH, HARTMANN group, Hamburg, Germany

**Keywords:** Hand hygiene dispenser, Alcohol-based hand rub, Point-of-care, Hand disinfection, Hand antisepsis

## Abstract

**Background:**

Hand hygiene is one of the most important hygiene measures to prevent healthcare-associated infections. Well-functioning hand rub dispensers are the foundation of hand hygiene but are often overlooked in research. As the point of origin for hand hygiene, dispensers not only promote compliance through ease of use, but also strongly influence the amount of hand rub used per disinfection. This work investigates how dispenser types and conditions affect dispensed volumes and usability.

**Methods:**

Data from 5,014 wall-mounted or point-of-care dispensers was collected from 19 German healthcare facilities during installation of an electronic hand hygiene monitoring system, including dispenser type and dispensed hand rub volumes. Of these dispensers, 56.2% were metal dispensers, and the majority (89.5%) were wall-mounted. For one hospital, 946 wall-mounted dispensers were analyzed in detail regarding pump material, damages, functionality, cleanliness, and filling levels.

**Results:**

Dispensed volumes varied across and within dispenser types, ranging from 0.4 mL to 4.4 mL per full actuation, with the largest volumes generally dispensed by plastic dispensers with a preset of 1.0 to 3.0 mL per actuation. In general, most dispensers dispense more hand rub per full actuation than specified by the manufacturer. When different types of dispensers are used within a healthcare facility, vastly different volumes can be dispensed, making reliable and reproducible disinfection difficult for healthcare workers. In the detailed analysis of 946 dispensers, 27.1% had cosmetic defects, reduced performance, or were unusable, with empty disinfectant being the most common reason. Only 19.7% of working dispensers delivered their maximum volume on the first full actuation.

**Conclusion:**

Even though several studies addressed the variability in dispensed volumes of hand hygiene dispensers, studies dealing with dispenser types and functionality are lacking, promoting the common but false assumption that different dispensers may be equivalent and interchangeable. Variability in dispensed volumes, coupled with frequent dispenser defects and maintenance issues, can be a major barrier to hand hygiene compliance. To support healthcare workers, more attention should be paid to ‘dispenser compliance’, selecting dispensers with similar volume ranges and proper maintenance.

## Background

Hand hygiene is considered the most important hygiene measure to prevent healthcare-associated infections (HAIs) [1]. However, hand hygiene compliance (HHC) remains a global issue despite decades of research and hundreds of available interventions [2]. HHC means using an effective disinfectant at the right moment correctly, which includes a sufficient volume, complete coverage of the hands, and sufficient rubbing time. While the indications for hand hygiene are determined by the ‘5 moments’ postulated by the World Health Organization (WHO) [3], the efficacy of a disinfectant is examined through standardized tests such as the European Standard EN 1500 [4]. While rubbing techniques often vary, and the rubbing time depends on the product used (e.g., by its alcohol concentration), the volume used must be sufficient to cover both hands completely. Generally, the applied volume of alcohol-based hand rub (ABHR) should not fall below 3 mL to ensure sufficient antimicrobial efficacy according to EN 1500 [5, 6]. At the same time, too large volumes can reduce the efficacy of hand disinfection through spillage [5, 6].

As convenience has been identified as a key driver of HHC [7, 8], recent research focused, for example, on the design of ergonomic workflows with optimal dispenser locations [9]. Correctly functioning dispensers represent the foundation of HHC, whereas empty or defective dispensers that dispense no or too little ABHR are a barrier to HHC and increase frustration with hand hygiene [10, 11]. To use enough ABHR for hand antisepsis, healthcare workers (HCWs) are often trained to use a specific number of pump actuations (usually 2–3). Yet, the amount of ABHR delivered per actuation is determined by the dispenser configuration and therefore varies between dispensers, thus making it difficult to transfer learned procedures between different dispensers. However, apart from examining optimal positions or minimum numerical requirements [12], dispensers and associated technical aspects such as dispensed volumes often remain unnoticed in compliance research and clinical practice. This is also reflected in the fact that responsibilities for dispensers are often not clearly defined. In some instances, each individual ward is tasked with refilling its own dispensers and responsibility for maintaining dispensers may fall between facility management and hygiene team, creating an accountability gap.

Although dispensers are often considered as equivalent and interchangeable in clinical practice, they can in fact have different configurations (e.g., metal vs. plastic housings/pumps), be used in different ways (e.g., manual vs. automatic), and dispense different volumes per actuation. Studies on different dispenser types and the influence of differences on correct hand hygiene are currently lacking.

Our aim was to obtain baseline information on the availability and functionality of ABHR dispensers in German healthcare facilities. To this end, we compiled dispenser information from several healthcare facilities collected during the installation of an electronic hand hygiene monitoring system. We focused on manual dispensers, which have been shown to be more reliable than automatic dispensers [13] and are more common in German healthcare facilities. In addition, a subset of dispensers from one of the included hospitals was examined in detail for their condition and functionality.

## Methods

This study is secondary research with data collected from healthcare facilities that have decided to install an electronic hand hygiene monitoring system on at least one ward. Information on ABHR dispensers was collected from March 2021 to March 2023 in 19 different German healthcare facilities, including 15 hospitals, three retirement/nursing homes, and one rehabilitation clinic. The healthcare facilities were in eight different federal states across Germany: five in Lower Saxony, four in North Rhine-Westphalia, three in Mecklenburg-Western Pomerania, two each in Baden-Württemberg and Saxony, and one each in Bavaria, Schleswig-Holstein and Brandenburg. The study includes a diverse range of hospitals: five facilities with 0-250 beds, four hospitals with 250–500 beds, another hospital providing 500–1000 beds, and two large hospitals with over 1000 beds. The retirement/nursing homes had 50 to 100 beds. Investigated dispensers were distributed throughout all areas of the healthcare facilities, including patient rooms, sanitary areas, waiting rooms, operating theaters, staff lounges and hallways.

The electronic hand hygiene monitoring system installed in these facilities was NosoEx^®^ (GWA Hygiene GmbH, Stralsund, Germany), which consisted of sensors and data hubs. Sensors were attached to existing wall-mounted and point-of-care (PoCs) dispensers, where they recorded the number of actuations and the volume of ABHR delivered per disinfection. Sensors on wall-mounted dispensers used magnetic detection and sensors on PoCs used pressure detection. Disinfection events were recorded anonymously by the sensor module and transmitted to the data hub via the NosoEx^®^ network. The data hub collected all data and transferred it to a database on a server of a certified data center.

**Fig. 1:**
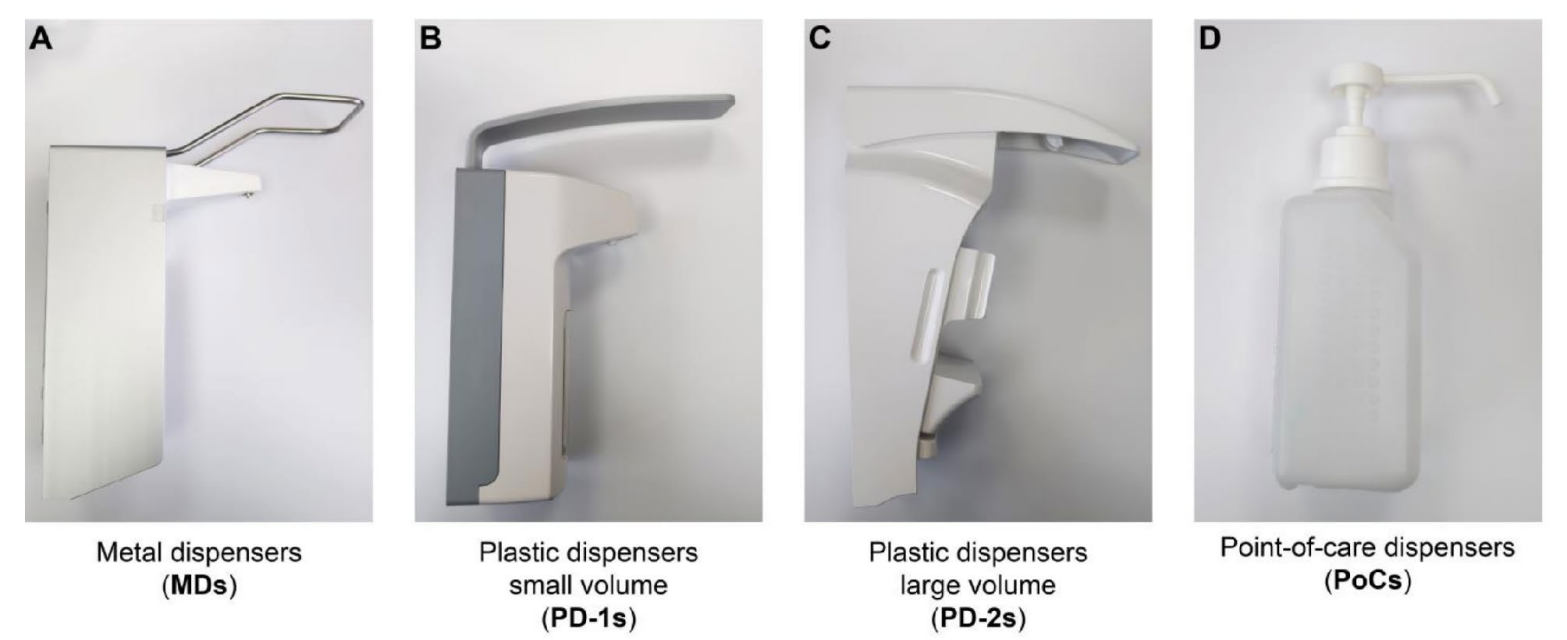
Examples of dispensers. Categorization of dispensers based on dispenser type and the volume ranges per actuation described in their specifications. The dispensers shown here are examples of the dispensers in each category: **(A)** metal dispensers (MDs), **(B)** plastic dispensers small volume (PD-1s), **(C)** plastic dispensers large volume (PD-2s), **(D)** point-of-care dispensers (PoCs)

During the installation of the electronic hand hygiene monitoring system, each sensor-equipped ABHR dispenser was individually and manually checked to obtain baseline values for its maximum dispensed volume. To this end, each dispenser was operated multiple times until it dispensed a constant volume; then the volume of five full actuations was collected in a measuring cylinder, with the average set as the volume per full actuation. As dispensers do not reliably deliver the same volume each time they are used, we averaged the volume dispensed over five actuations to provide a more accurate and reliable estimate of typical dispenser performance. Empty dispensers were refilled before this procedure. This was part of the installation process of the monitoring system. The maximum volumes measured during installation were used to adjust the system and improve the accuracy of volume estimation for each hand hygiene event, ensuring reliable monitoring of hand hygiene compliance. All data was anonymized prior to analysis. All dispensers contained liquid alcohol-based hand rubs, no gels or foams were used. In one hospital, 14 soap dispensers were equipped, which were excluded from this study. Overall, 5,014 dispensers from a range of manufacturers (Fig. 1) were included in this evaluation and classified into one of the following categories based on dispenser type and manufacturers’ specifications for the volume dispensed per actuation:

- Metal dispensers (MDs): wall-mounted, specified actuation volume 0.75–1.5 mL, metal housing fits euro-bottle, commonly known as ‘Eurodispenser’ (Fig. 1A).
- Plastic dispensers small volume (PD-1s): wall-mounted, specified actuation volume 0.5–1.5 mL, plastic housing fits euro-bottle (Fig. 1B).
- Plastic dispensers large volume (PD-2s): wall-mounted, specified actuation volume 1–3 mL, plastic housing fits euro-bottle (Fig. 1C).
- Mobile point-of-care dispensers (PoCs): mobile dispensers with single-use plastic pumps for flexible use at the point of care with various holders, e.g. mounted to patient beds or placed on desks (Fig. 1D).

In addition, 946 wall-mounted dispensers of one hospital were analyzed in more detail to gain a better understanding for product development. Here, the pump type (metal vs. plastic), damage to backplate and dispenser (plus functionality), dispenser cleanliness, and ABHR availability were recorded as well.

Data analysis was performed using Microsoft Excel version 2302 (Microsoft, Redmond, Washington, USA) and Python version 3.9 with libraries pandas (version 1.2.5), numpy (version 1.23.4), seaborn (version 0.12.1), matplotlib (version 3.6.0), and joypy (version 0.2.6).

## Results

### Dispenser types and volumes dispensed across facilities

The majority (2,820 − 56.2%) of all 5,014 investigated dispensers were MDs with a specified volume ranging from 0.75 to 1.5 mL (Table 1). Overall, 89.5% (4,488) of the dispensers analyzed were stationary (wall-mounted), whereas flexible PoCs made up only 10.5% of the dispensers evaluated. Dispensed volumes for single actuations ranged from 0.4 mL to 4.4 mL, with MDs generally dispensing the smallest volume (mean ± standard deviation (SD): 1.60 ± 0.14 mL; median: 1.59 mL). With 4.4 mL, the largest recorded volume per single actuation was documented for PoCs (Table 1).

**Table 1:** Overview of dispenser numbers, dispensed volumes, and defined volume ranges per dispenser type. Further specifications of dispenser types according to methods section. IQR, interquartile range; MD, metal dispenser; PD-1, plastic dispenser with a specified volume range of 0.5–1.5 mL; PD-2, plastic dispenser with a specified volume range of 1.0–3.0 mL; PoC, point-of-care dispenser; SD, standard deviation; V, volume

Type	*N* (%)	V_range_ [mL]	V_mean ± SD_ [mL]	V_median_ [mL]	V_mode_ [mL]	V_IQR_ [mL]	Specified V_range_ [mL]
**Metal dispensers** (MDs)	2,820 (56.2)	0.76–3.60	1.60 ± 0.14	1.59	1.60	0.23	0.75–1.5 mL
**Plastic dispensers 1** (PD-1s)	1,438 (28.7)	0.40–3.40	1.72 ± 0.14	1.73	1.73	0	0.5–1.5 mL
**Plastic dispensers 2** (PD-2s)	230 (4.6)	1.20–4.00	2.94 ± 0.56	3.00	3	0.3	1.0–3.0 mL
**Point-of-care dispensers** (PoCs)	526 (10.5)	1.48–4.40	2.67 ± 0.59	2.75	3	0.6	0.7–3.0 mL
**All**	**5**,**014 (100)**	**0.40–4.40**	**1.81** ± **0.48**	**1.73**	**1.73**	**0.17**	**0.5–3.0 mL**

**Fig. 2:**
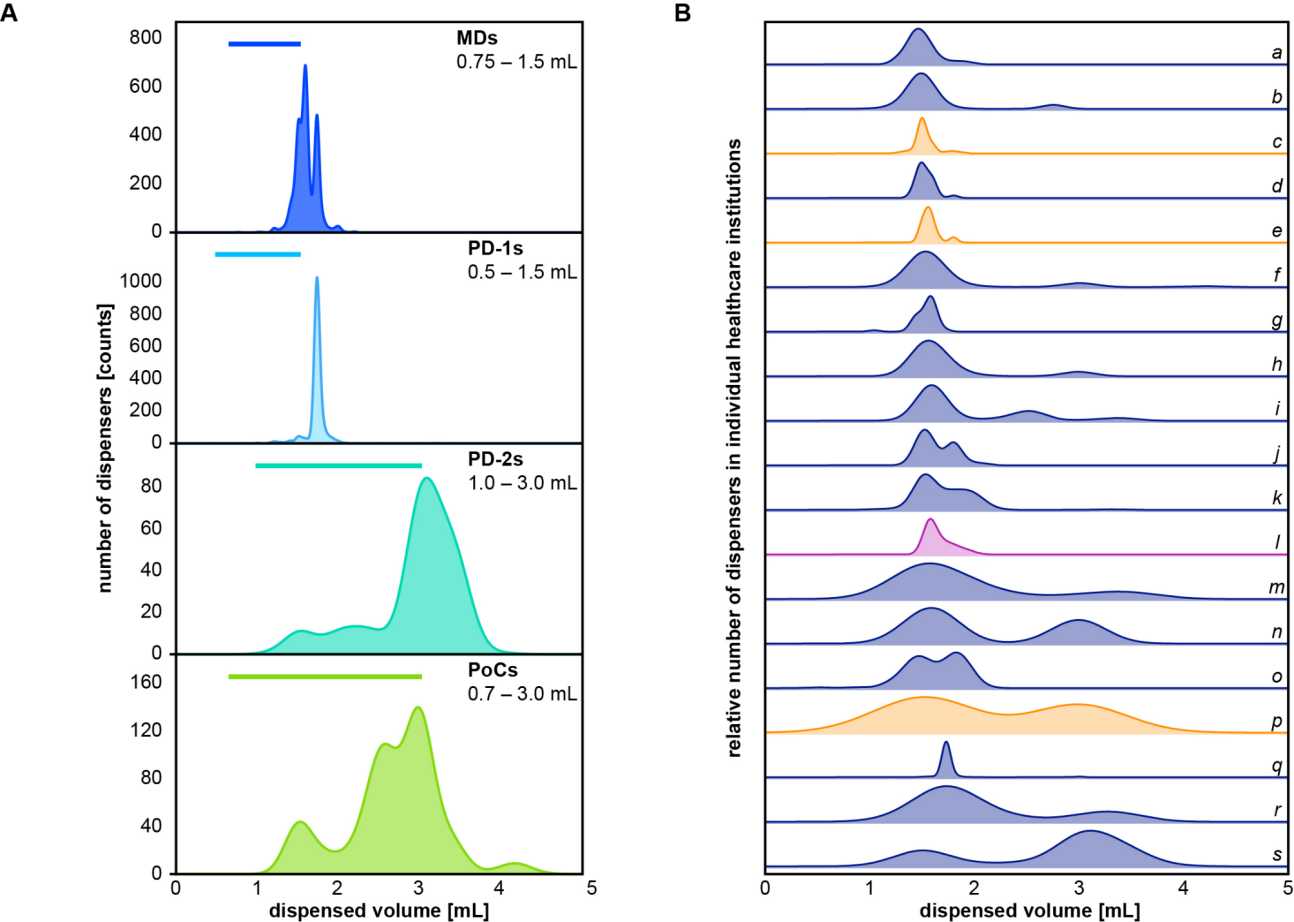
Disinfectant volumes dispensed by hand rub dispensers. Aggregated data from all 5,014 dispensers across 19 healthcare institutions. **(A)** Shown is the distribution of volumes dispensed on a single full actuation per dispenser type. Kernel density estimate plot of dispensed volumes. Horizontal lines indicate expected volume ranges for these dispensers based on the manufacturer information. X-axis = dispensed volume; y-axis = number of dispensers that dispense volume x. **(B)** Distributions of volumes dispensed within each individual healthcare facility. Kernel density estimate plot of dispensed volumes. Individual rows and lower-case letters indicate individual institutions. Hospitals are shown in blue, retirement/nursing homes in orange, and the rehabilitation clinic in purple. X-axis = dispensed volume; y-axis = relative number of dispensers that dispense volume x

The volume range dispensed by each dispenser type varied across all healthcare facilities (Fig. 2A). MDs had two distinct peaks, one at 1.6 mL and one at 1.73 mL. In contrast, PoCs only had a small peak at 1.5 mL, while most fell within a volume range of 2.2–3.6 mL. Compared to all other dispenser types, PD-1s delivered the most constant volume, which was 1.73 mL in most dispensers. Volumes of PD-2s mainly ranged from 2.7 to 3.8 mL. Overall, both PD-1s and MDs were very consistent in comparison with PoCs and PD-2s, which dispensed a range of different volumes. Most dispensers delivered volumes exceeding their specified range.

Consistent dispensed volumes are crucial for reliable hand antisepsis, as HCWs are often trained to use a specific number of actuations. Yet, even within individual healthcare facilities, dispensers gave vastly different volumes (Fig. 2B). In several facilities (for example Fig. 2B m, p, or s), volumes ranged from below 1 mL to over 4 mL. In most facilities, the majority of dispensers delivered between 1.5 and 2 mL per full actuation (for example Fig. 2B a, c, or i), while some dispensers delivered around 3 mL per actuation in five facilities (Fig. 2B f, h, n, p, s).

### Dispenser functionality and impact of pump material on volume and reliability

We analyzed 946 wall-mounted dispensers from a single hospital in more detail, of which 273 (28.9%) had at least one problem (Table 2). Problems ranged from cosmetic defects (dirt, cracks on external surfaces) to reduced performance (complete actuation not possible) to the inoperability of the dispenser (disinfectant empty). In particular, the impact of dirt on pump functionality was difficult to assess. Of the 946 dispensers, 834 could be made operational during the course of the installation of the hand hygiene monitoring system. Of these, only 164 (19.6%) dispensers delivered the full volume on the first full actuation (Table 3).

**Table 2:** Defects and deficits of hand rub dispensers. Proportion of dispensers that were not fully functional due to damage or other problems. Data refer to the subset of 946 wall-mounted dispensers in a single hospital. Dispensers may have more than one problem at a time and may therefore be counted in more than one category

Problem	*N*	%
ABHR empty	120	12.68
Pump/dispenser soiled	72	7.61
Backplate damaged	68	7.19
Dispenser damaged	39	4.12
Full stroke not possible	22	2.33
**All dispensers**	**946**	**100.00**

**Table 3:** Pre-actuations required to achieve complete pump volume. Pre-actuations required to pump ABHR to deliver the full volume of one full actuation. None means that the full volume was delivered on the first actuation and no pre-actuation was required. Data refer to a subset of 834 of the 946 wall-mounted dispensers that could be made operational

Pre-strokes to full volume	*N*	%
None	164	19.66
1	220	26.38
2	370	44.36
3	65	7.79
4	8	0.96
5	4	0.48
6	1	0.12
7	0	0.00
8	1	0.12
9	1	0.12
**Overall**	**834**	**100.00**

**Fig. 3:**
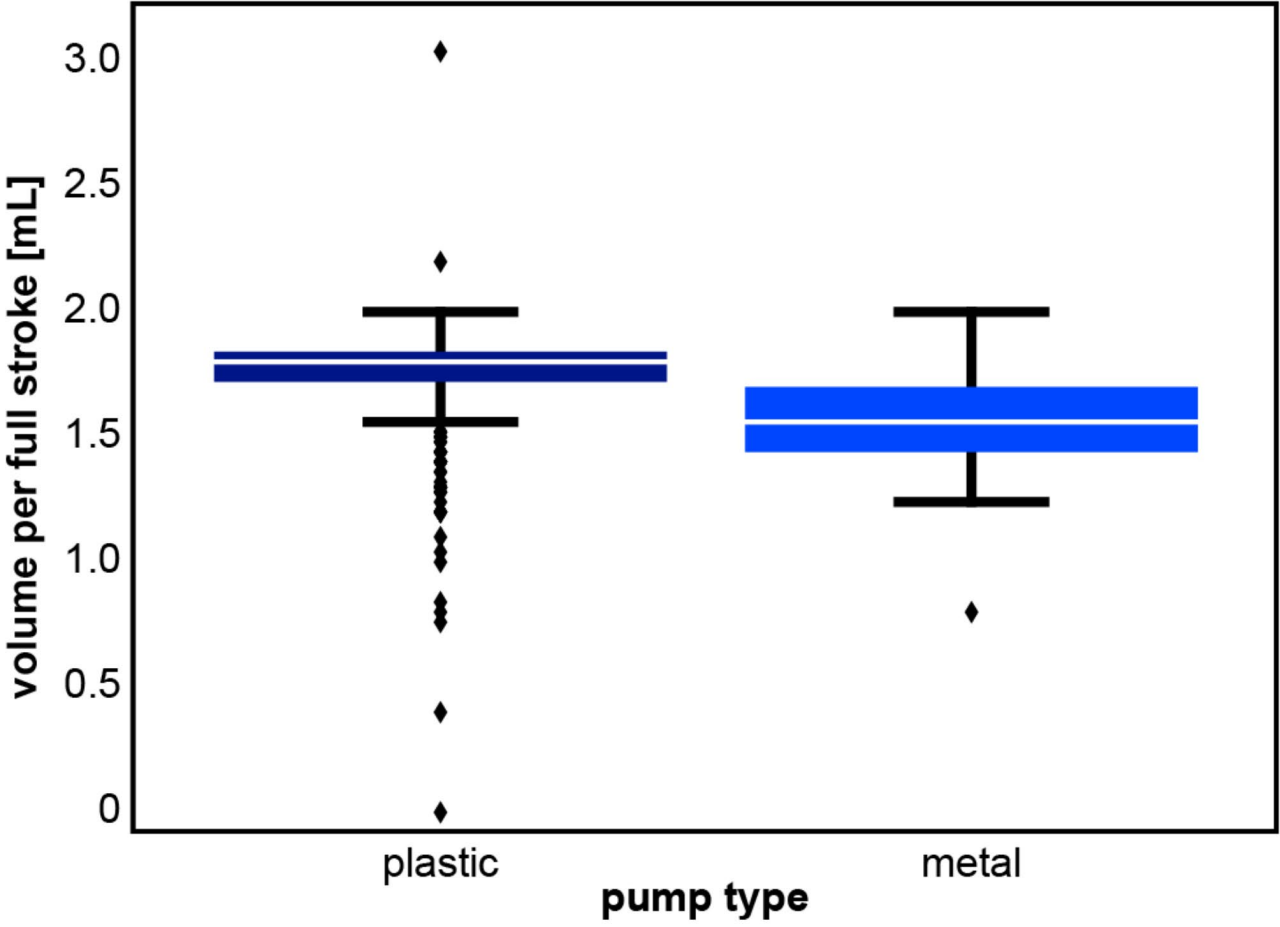
Influence of pump material on dispensed disinfectant volumes. Volume per pump actuation depending on the material of the pumps (plastic vs. metal). Median: plastic 1.8 mL, metal 1.56 mL. Standard deviation: plastic/metal each 0.18 mL. Variance: plastic/metal each 0.03 mL

The pump in a dispenser affects both the volume dispensed and the reliability. Of the 946 dispensers, 780 (82.5%) had plastic pumps and 140 (14.8%) had metal pumps, while no information was documented for 26 (2.7%). Overall, metal pumps dispensed a more variable volume than plastic pumps (Fig. 3). Outliers were much more frequent with plastic pumps, which are not designed for long-term use with multiple bottles. In general, the variance was similar for metal and plastic pumps, indicating comparable reliability regarding the maximum volume (Fig. 3). Generally, volumes dispensed by plastic pumps (mean ± SD: 1.76 ± 0.18 mL, median: 1.80 mL) were larger than those dispensed by metal pumps (mean ± SD: 1.57 mL ± 0.18, median: 1.56 mL).

## Discussion

Our study reveals several key insights into the functionality of ABHR dispensers in clinical settings. More than a quarter of dispensers had problems such as empty bottles, dirt, minor damage, or hard-to-use pumps, affecting their usability. More than 80% of functional dispensers required multiple actuations to deliver the full volume of ABHR, with some needing up to nine pre-actuations. The volume delivered per actuation varied significantly between dispenser types, ranging from 0.4 mL to 4.4 mL. PoC dispensers, in particular, showed considerable inconsistency.

Although it is undisputed that correct hand hygiene at the indicated moments is one of the most important strategies for reducing HAIs [14, 15], HHC remains a global issue. Improving HHC reduces infection rates and the associated morbidity, mortality, length of stay, and costs [16, 17]. Well-functioning ABHR dispensers form the foundation of hand hygiene, but research has so far mostly focused on optimal numbers and positioning. Accordingly, there is strong evidence showing that highly visible and accessible dispensers can increase HHC [18]. While innovations such as electronic monitoring systems to collect data on hand hygiene behaviour are bringing hand rub dispensers into the spotlight [19, 20], studies on the basic functionality of dispensers are still lacking.

The recently published ‘ISO 23447:2023 Healthcare organization management — Hand hygiene performance’ [21], which specifies requirements for hand hygiene in the healthcare sector worldwide, includes requirements for hand hygiene dispensers: dispensers must be sealed and hygienically intact, prevent direct contact with the product, dispense the specified volume consistently over the lifetime of the dispenser, have cleaning and reprocessing guidelines, prohibit refillable systems and be replaced when damaged or faulty. This norm also recommends the establishment of an operations and maintenance plan and provides specific recommendations and requirements for the maintenance of the dispensers. Facility management should oversee the installation, maintenance, ordering and compliance of hand rub dispensers, while unit staff are responsible for reporting problems for timely action. The majority of the dispensers examined here, namely MDs, PD-1s and many of the PD-2s, are non-sealed, refillable systems, and the fact that about a quarter of the dispensers were inoperable suggests that proper maintenance plans were not in place or that maintenance was not performed regularly.

Several studies have shown that many dispensers fail to consistently dispense the specified volume throughout their lifetime and that dispensed volumes can vary due to periods of non-use and depending on liquid levels [22–24]. For example, Bánsághi and Haidegger tested 13 different ABHR dispensers with both liquids and gels, using a standardized test protocol [25]. They observed that periods of non-use, even as short as 5 min, resulted in reduced ABHR volumes [25], requiring pre-actuations, similar to those we observed (Table 2), to restore delivery of the full volume. This phenomenon is less noticeable with viscous formats such as gels than with liquids [23]. For PoCs, this is often caused by incomplete pump actuations that do not allow for pressure equalization, creating a negative pressure in the bottle and allowing ABHR to be drawn back into the bottle. Even when fully functional, some of the dispensers deliver too little ABHR, as was the case with some PD-1s, which delivered only 0.4 mL per actuation. The low volume of disinfectant dispensed results in inadequate hand coverage, particularly for HCWs with larger hands [6]. Given the importance of convenience for hand hygiene compliance [7, 8], the fact that most HCWs find it difficult to estimate ABHR volumes [23, 25], and the reality that ABHR volumes per disinfection are typically less than 3 mL, HCWs are likely to accept a lower ABHR volume [20, 26]. High volumes of 3.5 mL or more are also inconvenient because of the potential for spillage and prolonged drying time [5], we have observed volumes of up to 4 mL per application (Fig. 2A). Poorly maintained dispensers can further have a serious impact on HHC [11]. Around a quarter of the dispensers examined in detail had at least one problem that made them difficult to use or unusable. While an empty bottle of disinfectant renders a dispenser unusable, dirt, minor damage, or hard-to-use pumps can reduce delivered volumes. We only looked at manual dispensers, which are generally considered more reliable than automatic dispensers [13]. However, even among manual dispensers, a significant proportion do not function properly [22], so we assume that the malfunction rate would have been even higher with automatic dispensers.

Although dispensers often do not deliver their full volume the first time they are activated, our data show that the maximum volume delivered is usually higher than specified. This is in contrast to the results reported by Bánsághi and Haidegger who tested 13 dispensers and found that most dispensed less than the specified 1.5 mL [25]. In our dataset, even the median and mean volumes of MDs and PD-1s were higher than their respective specified maximum. However, the volumes dispensed varied considerably between different dispenser types, resulting in some facilities reporting volumes ranging anywhere between 0.4 mL and 4.4 mL after a single actuation. This may be due to different dispenser types being used, with PoCs proving to be particularly inconsistent. Accordingly, training HCWs to use a specific ABHR volume may be challenging, especially if they are expected to learn to use a specific number of dispenser actuations. While exclusive use of MDs and PD-1s would result in more consistent volumes, the inconsistency of single-use PoC pumps remains an issue. Yet, PoCs are more flexible and can easily be positioned close to the point of care where they can positively affect HHC. Accordingly, positioning ABHR dispensers near patient beds directly in the field of view has been shown to increase HHC [27], and providing flexibly mountable dispensers as part of a multimodal intervention can even help improve HHC before aseptic procedures [28].

In addition to the differences in dispensed volume between the four dispenser types, dispensed volumes can vary even within dispensers of one type when different pumps are used, as demonstrated by the two peaks for MDs in Fig. 2A, which derive most likely from plastic (1.73 mL) and metal pumps (1.6 mL) (Figs. 2A and 3). Comparing the reliability of both materials, metal and plastic pumps had a similar variance in dispensed volumes. Plastic pumps are single-use and require frequent replacement. When used only once according to their intended use, they may be as reliable as metal pumps, which is not the case when they are erroneously used over prolonged periods. In contrast, metal pumps designed for reuse must be reprocessed regularly, which is why they are not recommended by ISO 23447:2023 [21]. They may be more reliable in delivering ABHR but can become a source of microbial contamination when not reprocessed properly.

Addressing the current challenges associated with hand hygiene dispensers in hospitals will require a multi-faceted approach based on research, dispenser design, and organizational frameworks. Future research should focus on understanding how factors such as proximity to functional dispensers influence user behaviour, particularly in terms of timely reporting or addressing non-functional dispensers. As Bánsághi and Haidegger emphasized [25], standardized testing of dispensers is crucial to ensure consistent and reliable performance. Without standardized evaluations, it will remain difficult to determine which dispensers reliably meet the high demands of healthcare environments. The design of dispensers should always allow for a quick assessment of fill levels, and responsibility for dispensers should be clearly assigned. The recently published ISO 23447:2023 provides a solid framework for defining the allocation of responsibility [21].

### Limitations

All data are snapshots collected during the installation of the electronic hand hygiene monitoring system. Wall-mounted dispensers were overrepresented in the dataset since they were more often equipped with the electronic hand hygiene monitoring system, while only 10.5% of all investigated dispensers were PoCs. Accordingly, all data derive from healthcare facilities that have actively chosen to use an electronic hand hygiene monitoring system. These are likely to be facilities where hygiene is important, or a strong hygiene team is in place. This could introduce bias since the proportion of properly functioning dispensers may be even lower in other facilities not studied here. Beyond that, neither automatic dispensers nor pocket bottles were included; however, pocket bottles in particular could be used at the point of care and compensate for the small number of PoCs. In some cases, when dispensers—particularly MDs—appeared to be functioning properly, a default value of 1.5 mL per actuation was assumed, although the actual volume could have been between 1.4 and 1.6 mL, creating uncertainty in the data. In addition, damage or dirt on dispensers may have led to changes in dispensed volumes. The maintenance status was only analyzed for one hospital, which might not be representative of other healthcare facilities. Due to the secondary study design, there are other limitations to the study, such as whether measures were taken to address compliance with reprocessing and maintenance of dispensers, or whether any further action was taken to address the observed variation in dispensed volumes within the hospital.

## Conclusions

Since convenient hand hygiene is only possible through well-functioning dispensers, our results suggest that more attention should be paid to ‘dispenser compliance’, which includes choosing dispensers with comparable actuation volumes as well as proper dispenser maintenance (e.g., regular ABHR refilling, cleaning, checking) and proper use (full pump actuations). In addition, the combination of reliable dispensers with tailored training makes it easier for HCWs to intuitively use the correct volume for hand hygiene. Although there are many different types of dispensers, they are not all the same. The choice of dispenser should ultimately be based on its reliability in delivering the appropriate volume of ABHR to HCWs.

## Data Availability

All data generated or analysed during this study are included in this published article.

## Abbreviations

ABHR: Alcohol-based hand rub
HAIs: Healthcare-associated infections
HCWs: Healthcare workers
HHC: Hand hygiene compliance
MDs: Metal dispensers
PD: 1s-plastic dispensers small volume (0.5-1.5 mL)
PD: 2s-plastic dispensers large volume (1-3 mL)
PoCs: Point-of-care dispensers

## References

[1] Hauer T, Dettenkofer M. Epidemiologie Und Prävention Von Nosokomialen Infektionen. Pädiatrie. 2014;25:820–41.

[2] Ikuta KS, Swetschinski LR, Robles Aguilar G, Sharara F, Mestrovic T, Gray AP et al. Global mortality associated with 33 bacterial pathogens in 2019: a systematic analysis for the Global Burden of Disease Study 2019. Lancet. 2022;400:2221–2248. https://linkinghub.elsevier.com/retrieve/pii/S0140673622021857 10.1016/S0140-6736(22)02185-7 PMC9763654 36423648

[3] World Health Organization. WHO guidelines on hand hygiene in health care. 2009. 10.1086/600379 19508124

[4] DIN EN 1500–2013. Chemical disinfectants and antiseptics - Hygienic handrub - Test method and requirements (phase 2/step 2). 2013.

[5] Voniatis C, Bánsághi S, Ferencz A, Haidegger T. A large-scale investigation of alcohol-based handrub (ABHR) volume: hand coverage correlations utilizing an innovative quantitative evaluation system. Antimicrob Resist Infect Control. 2021;10:49. 33678183 10.1186/s13756-021-00917-8 PMC7937362

[6] Voniatis C, Bánsághi S, Veres DS, Szerémy P, Jedlovszky-Hajdu A, Szijártó A, et al. Evidence-based hand hygiene: liquid or gel handrub, does it matter? Antimicrob Resist Infect Control. 2023;12:12. 36782305 10.1186/s13756-023-01212-4 PMC9926746

[7] Kirk J, Kendall A, Marx JF, Pincock T, Young E, Hughes JM et al. Point of care hand hygiene—where’s the rub? A survey of US and Canadian health care workers’ knowledge, attitudes, and practices. Am J Infect Control. 2016;44:1095–1101. 10.1016/j.ajic.2016.03.005 10.1016/j.ajic.2016.03.005 27178035

[8] Zhao Q, Yang MM, Huang Y-Y, Chen W. How to make hand hygiene interventions more attractive to nurses: a discrete choice experiment. PLoS ONE. 2018;13:e0202014. 30092024 10.1371/journal.pone.0202014 PMC6084975

[9] Dick A, Sterr CM, Dapper L, Nonnenmacher-Winter C, Günther F. Tailored positioning and number of hand rub dispensers: the fundamentals for optimized hand hygiene compliance. J Hosp Infect. 2023;141:71–9. 37660889 10.1016/j.jhin.2023.08.017

[10] Armstrong-Novak J, Juan HY, Cooper K, Bailey P. Healthcare Personnel Hand Hygiene Compliance: are we there yet? Curr Infect Dis Rep. 2023;1–7. 10.1007/s11908-023-00806-8 PMC10213575 37361491

[11] Bezpalko O, Ponnala S, Won JC. All hands on deck: sustaining Improved Hand Hygiene Compliance in the operating room. Ergon Des. 2022;30:4–10.

[12] Kuster S, Roth JA, Frei R, Meier CA, Dangel M, Widmer AF. Handrub dispensers per acute care hospital bed: a study to develop a new minimum standard. Antimicrob Resist Infect Control. 2021;10:93. 34134772 10.1186/s13756-021-00949-0 PMC8206889

[13] Roth JA, Batzer B, Hug BL, Widmer AF. Defect Rates in Touchless Versus Mechanical Hand Hygiene Dispensers. Infect Control Hosp Epidemiol. 2018;39:359–60. 29363435 10.1017/ice.2017.306

[14] Cardo D, Dennehy PH, Halverson P, Fishman N, Kohn M, Murphy CL, et al. Moving toward elimination of healthcare-associated infections: a call to action. Infect Control Hosp Epidemiol. 2010;31:1101–5. 20929300 10.1086/656912

[15] Ranji SR, Shetty K, Posley KA, Lewis R, Sundaram V, Galvin CM et al. Closing the Quality Gap: A Critical Analysis of Quality Improvement Strategies (Vol. 6: Prevention of Healthcare–Associated Infections) [Internet]. Agency for Healthcare Research and Quality (US), Rockville (MD); 2007. http://europepmc.org/books/NBK43982 20734530

[16] Pittet D, Hugonnet S, Harbarth S, Mourouga P, Sauvan V, Touveneau S, et al. Effectiveness of a hospital-wide programme to improve compliance with hand hygiene. Infect Control Programme Lancet. 2000;356:1307–12. 10.1016/s0140-6736(00)02814-2 11073019

[17] Stewart S, Robertson C, Pan J, Kennedy S, Haahr L, Manoukian S, et al. Impact of healthcare-associated infection on length of stay. J Hosp Infect. 2021;114:23–31. 34301393 10.1016/j.jhin.2021.02.026

[18] Cure L, Van Enk R. Effect of hand sanitizer location on hand hygiene compliance. Am J Infect Control. 2015;43:917–921. 10.1016/j.ajic.2015.05.013 10.1016/j.ajic.2015.05.013 26088769

[19] Scheithauer S, Bickenbach J, Heisel H, Fehling P, Marx G, Lemmen S. Do WiFi-based hand hygiene dispenser systems increase hand hygiene compliance? Am J Infect Control. 2018;46:1192–1194. 10.1016/j.ajic.2018.03.026 10.1016/j.ajic.2018.03.026 29779691

[20] Senges C, Herzer C, Norkus E, Krewing M, Mattner C, Rose L, et al. Workflows and locations matter - insights from electronic hand hygiene monitoring into the use of hand rub dispensers across diverse hospital wards. Infect Prev Pract. 2024;6:100364. 38601127 10.1016/j.infpip.2024.100364 PMC11004075

[21] ISO International Standard. ISO/CD2 23447:2022 Healthcare organization management — hand hygiene performance and compliance. Switzerland, 2022.

[22] Kohan C, Ligi C, Dumigan DG, Boyce JM. The importance of evaluating product dispensers when selecting alcohol-based handrubs. Am J Infect Control. 2002;30:373–5. 12360146 10.1067/mic.2002.125586

[23] Bánsághi S, Soule H, Guitart C, Pittet D, Haidegger T. Critical reliability issues of common type alcohol-based Handrub dispensers. Antimicrob Resist Infect Control. 2020;9:90. 32571388 10.1186/s13756-020-00735-4 PMC7310242

[24] Arbogast JW, Smith NM, Bansaghi S, Chen N, Neal TB, McNulty JJ, et al. Dosing matters–consistency analysis of Touch-Free Automatic Hand Hygiene dispensers. Open Forum Infect Dis. 2023;10(Suppl 2):ofad500–1268.

[25] Bánsághi S, Haidegger T. Standardized test method to assess the functions and working characteristics of Handrub dispensers. Acta Polytech Hungarica. 2023;20:197–217.

[26] Scheithauer S, Schwanz T, Koch A, Häfner H, Krizanovic V, Lemmen S. Steigerung Des Verbrauchs an Hände-Desinfektionsmittel Nach Einführung berührungsfreier Desinfektionsmittelspender. Hyg + Medizin. 2011;36:496–8.

[27] Birnbach DJ, Nevo I, Scheinman SR, Fitzpatrick M, Shekhter I, Lombard JL. Patient safety begins with proper planning: a quantitative method to improve hospital design. Qual Saf Heal Care. 2010;19:462–5. 10.1136/qshc.2008.031013 20584700

[28] Aghdassi SJS, Schröder C, Lemke E, Behnke M, Fliss PM, Plotzki C, et al. A multimodal intervention to improve hand hygiene compliance in peripheral wards of a tertiary care university centre: a cluster randomised controlled trial. Antimicrob Resist Infect Control. 2020;9:113. 32682429 10.1186/s13756-020-00776-9 PMC7368705

